# Characteristics of High Risk People with Cardiovascular Disease in Chinese Rural Areas: Clinical Indictors, Disease Patterns and Drug Treatment

**DOI:** 10.1371/journal.pone.0054169

**Published:** 2013-01-18

**Authors:** Xiaolin Wei, Guanyang Zou, Jia Yin, John Walley, Biao Zhou, Yunxian Yu, Linwei Tian, Kun Chen

**Affiliations:** 1 School of Public Health and Primary Care, Chinese University of Hong Kong, Hong Kong, China; 2 Nuffield Centre for International Health and Development, University of Leeds, Leeds, United Kingdom; 3 School of Public Health, Zhejiang University, Hangzhou, China; Maastricht University Medical Center, The Netherlands

## Abstract

**Background and Aims:**

Current cardiovascular disease (CVD) prevention is based on diagnosis and treatment of specific disease. Little is known for high risk people with CVD at the community level. In rural China, health records of all residents were established after the recent health reforms. This study aims to describe the characters of the rural population with high CVD risk regarding their clinical indicators, disease patterns, drug treatment and adherence.

**Methods and Results:**

17042 (87%) of all the 19500 rural residents in the two townships had valid health records in 2009. We employed a validated tool, the Asian Equation, to screen 8182 (48%) resident health records of those aged between 40–75 years in 2010. Those who were identified with a CVD risk of 20% or higher were selected for a face-to-face questionnaire survey regarding their diagnosed disease and drug treatment. 453 individuals were identified as high risk of CVD, with an average age of 53 years, 62% males, 50% smoking rate and average systolic blood pressure of 161 mmHg. 386 (85%) participated in the survey, while 294 (76%) were diagnosed with and 88 (23%) were suspects of CVD, hypertension, diabetes or hyperlipidaemia. 75 (19%) took drug regularly and 125 (32%) either stopped treatment or missed drugs. The most often used drugs were calcium channel blockers (20%). Only 2% used aspirins and 0.8% used statins. The median costs of drugs were 17 RMB (USD2.66) per month.

**Conclusion:**

The majority of the high risk population in our setting of rural China had already been diagnosed with a CVD related disease, but very few took any drugs, and less still took highly effective drugs to prevent CVD. A holistic strategy focused on population with high risk CVD and based on the current China public health reform is suggested in the context of primary care.

## Introduction

Cardiovascular disease (CVD) is the leading cause of death in China, accounting for 38% of total mortality [1]. China’s rapid economic development has resulted in an increase in non-communicable chronic diseases. The lifestyle of Chinese populations has changed dramatically, mirroring that of more affluent countries with sedentary lifestyles, higher consumption of meat and changing diets, which are high in salt, sugar and fats. In 2006, China was estimated to have 160 million people with hypertension, 160 million with hyperlipidaemia, 20 million with diabetes and 200 million who were overweight [1]. The reported mortality rates of stroke had increased at a faster rate in rural areas compared to urban areas between 1990 and 2001, while the reported stroke mortality rates fluctuated in both rural and urban areas from 2001 to 2004 [2]. Compared with the Western population, Chinese population has a higher proportion of reported haemorrhagic stroke [3]. Some studies reported that the variation of the proportions of haemorrhagic stroke was also large between the north and south China [4]. Haemorrhagic stroke incidence rates in rural China may be much higher than reported rates due to the underreported mortality cases of haemorrhagic stroke and the less use of key diagnostic tools such as computed tomography (CT) and magnetic resonance imaging (MRI) scanning in stroke patients [2], [5]. It is predicted that annual CVD events in China will increase by 23% from 2010 to 2030. This will result in an additional 21 million CVD events and 7.7 million CVD deaths [6]. It is predicted that China will lose US$558 million in national income as a result of CVD and diabetes over the next 10 years [7].

The overall risk prediction strategy considers all risk factors in a person and calculates the overall CVD risk to inform treatment decisions. It is advocated that separate management guidelines for hypertension and diabetes should be replaced by integrated CVD risk management guidelines, because individuals with given blood pressure may have a 20-fold variation in CVD risk due to other factors such as smoking, age, body mass index and diabetes status [8]. The risk assessment tool needs to be revalidated in specific populations, because systematic reviews found that some tools under-predicted risk in high risk groups and over-predicted risks in low risk groups among Chinese population [9].

In this study, we applied the Asian Equation to assess the absolute CVD risk of a rural population in China. The Asian Equation was derived based on population cohorts joined in the Asia Pacific Cohort Studies Collaboration, i.e., a total of 172,077 individuals who were followed up for 4–20 years in Japan, Korea and Singapore [10]. The equation had fewer variables for CVD risk prediction compared with the Framingham one to fit for the low information availability in developing countries. It calculates a person’s percentage risk of having any CVD event in the next 8 years; based on his/her gender, age, systolic blood pressure, total cholesterol and smoking status. The performance of the Asian Equation has been validated using six Chinese cohorts of 25,682 individuals with up to 12 years follow-up in both urban and rural China, and was compared with a recalibrated Framingham Equation for Chinese population [10]. The outcome of interest was any cardiovascular event, defined as cardiovascular death, non-fatal myocardial infarction or non-fatal cerebrovascular events. This showed a 11% and 10% overestimation of CVD events in men and women compared with 276% and 102% of overestimation in men and women by the recalibrated Framingham equation [10]. Its discrimination ability is similar to other CVD risk prediction equations [11], [12]. Currently, the Asian Equation is the best available tool that can effectively predict both ischaemic and haemorrhagic CVD events in Chinese population, while other tools can only predict ischaemic CVD events but not haemorrhagic stroke [11], [12].

Systematic reviews suggest that using two antihypertensive drugs simultaneously will reduce the risk of stroke by 63% and ischaemic disease by 46% [13], while statins will reduce the risk by 60% and 17% respectively [14]. The combined effects of three antihypertensive drugs, plus a statin and aspirin would reduce by more than 88% ischaemic heart disease events and by 80% stroke events [15]. Furthermore folic acid reduces the risk of stroke by 18% [16] in hypertensive patients with high concentration of hyperhomocysteinemia; and up to 75% of hypertensive patients have high hyperhomocysteinemia [17], [18]. Food in China is not yet fortified with folic acid. Two recent trials of the fixed-dose combination (known as polypill) successfully reduced systolic blood pressure by 6–7 mmHg, which translates as around 60–75% CVD risk reduction in the general population over 45 years old [19], [20]. Therefore, we examined use of antihypertensive drugs, statin, aspirin and folic acid among the high risk people with cardiovascular disease in Chinese rural areas.

With the health reform launched in China, one strategy was to improve public health service at the primary care level [21], and to set up resident health records containing social and bio indicators of all residents in the community. This enables the use of risk assessment tools. In this study, we employed the Asian Equation to identify population with high CVD risk from the health records of two townships in rural Zhejiang China. The study aims to describe the characters of the rural population with high CVD risk regarding their blood pressures, lipid profile, disease patterns, drug treatment and adherence. Specific objectives of the study are: (1) to screen and identify people with high CVD risk using health records of rural residents collected in routine primary care settings; (2) to find out disease patters and risk factors for those people at high CVD risk; and (3) to assess whether their diseases and risk factors have been treated properly. The paper also discusses the possibility of launching a CVD risk based treatment strategy in rural China.

## Methods

### Setting

Zhejiang is located in the east of China, with a GDP per capita of USD7690, 1.8 times of the national average. We selected two townships with a population of 19,500 in central Zhejiang. The two townships were of average economic development in Zhejiang, and presented a typical rural community.

### Data Collection

All rural residents in the study area have participated in the New Cooperative Medical Scheme (NCMS), i.e., the rural health insurance scheme in China. Free health checks are provided as a benefit to enrolled residents annually and the results are stored in health records. We employed all routine health checks collected between July and December 2009 in the studied area. The Asian Equation was applied to the database of resident health records in the two rural townships for those between 40–75 years old. The Asian Equation was validated for those below 75 years old [10], while UK NICE guidelines suggest targeting CVD prevention for people aged above 40 [22], [23]. Those who were identified with a CVD disease risk of 20% or higher were selected for a face to face questionnaire survey regarding their diagnosed disease, current treatment with drugs, adherence and costs of drugs, if any. Diagnosis of CVD, hypertension, diabetes and hyperlipidaemia were made according to China national guidelines [5] and recorded in the resident health records. Variables regarding diagnosis, gender, age, body mass index, blood pressures and total cholesterol were extracted from the resident health records. Smoking was defined as current smokers with at least one cigarette a day regardless of the duration of smoking, which was in line with the definitions of smoking rate in the Asian Equation [10]. Family doctors of the township hospitals were trained and collected the data in 2010. Ethics approval was obtained from the Zhejiang University ethical committee. Written informed consent forms were collected from all participants.

### Analysis

Data was checked, coded and double entered into a database using Epidata and then analyzed using SPSS 14.0 (Chicago, USA). Descriptive analysis including independent t-test, chi-square test, and non-parametric data comparison were used when appropriate. P = 0.05 was quoted consistently as the statistical significance level.

## Results

In the studied area, 17,042 (87%) of all the 19,500 residents had valid health records. The proportions of males were similar between residents with health records and all the residents (50.6% vs. 51.8%). Proportions of the age bands of 0–14, 15–59 and ≥60 were 14.0%, 71.9% and 14.1% in all the residents and 10.0%, 74.1% and 15.9% in residents with health records, respectively. Although residents with health records were slightly older compared with all the residents, the statistical difference may be drawn due to the large numbers in each cell of the chi-square table. Of the two townships, 8182 residents (48%) between 40 and 75 years old were assessed (Table 1). The Asian Equation identified 453 residents having a CVD risk of 20% or higher in the next 8 years, accounting for 5.5% of the residents between 40 to 75 years old, or 2.3% of the total population. The median risk of CVD in the high risk population was 26.4%, which was 7.3 times the median risk of the low risk population and 6.9 times of the general population. The average age of the high risk population was 69 years old, significantly higher than that of the population with CVD risk less than 20%. The high CVD risk population had significantly more males than the low risk population. The smoking rate of the population with high CVD risk was 1.8 times that of the low risk population. Compared with the low risk population, the high CVD risk population had significantly higher average systolic blood pressure, diastolic blood pressure and total cholesterol level. Of people at high CVD risk, 21.9% had fasting blood glucose over 6.1 mmol/L, indicating impaired fasting glycaedemia or diabetes. This proportion was significantly lower among the low risk population. These differences between high and low risk populations for BP and cholesterol remained when analysed separately for men and for women.

**Table 1:** Cardiovascular (CVD) risks factors of the 8182 rural residents aged 40 to 75 years old in Zhejiang, China.

	Population with high risk of CVD			Population with low risk of CVD			General population		
	Male	Female	Sub-total	Male	Female	Sub-total	Male	Female	Sub-total
Number	280 (62)^a^	173 (38)	453	3562(46)	4167 (54)	7729	3842 (47)	4340 (53)	8182
Age	68	70	69^b^	53	53	53	54	54	54
Systolic blood pressure (mmHg)	155.1	170.0	160.8^c^	120.6	122.4	121.6	123.0	124.2	123.7
Diastolic blood pressure (mmHg)	86.5	90.5	88.1^d^	77.4	76.4	76.9	77.9	76.9	77.4
Fasting blood glucose(FBG) (mmol/L)	5.6	5.8	5.7^e^	5.5	5.6	5.6	5.5	5.6	5.6
Number and proportionof people with FBG≧6.1 mmol/L	52 (18.6)	47 (27.2)	99 (21.9)^f^	620 (17.4)	663 (15.9)	1283 (16.6)	672 (17.5)	710 (16.4)	1382 (16.9)
Total cholesterol level (mmol/L)	4.8	4.9	4.8^g^	4.5	4.6	4.6	4.5	4.6	4.6
BMI	22.0	22.8	22.3	22.4	22.7	22.5	22.4	22.7	22.5
Smoking rate (%)	207 (73.9)	9 (5.2)	216 (47.7)^h^	2002 (56.1)	79 (1.9)	2079 (26.9)	2209 (57.4)	88 (2.0)	2297 (28.1)
Median of CVD risk (%)	26.5	25.9	26.4^i^	4.3	2.9	3.6	4.8	3.1	3.8

Significantly higher than the population with low risk of CVD: ^a^(χ^2^ = 42.478, P<0.001), ^b^(t = 38.733, P<0.001), ^c^(t = 44.737, P<0.001), ^d^(t = 21.040, P<0.001), ^e^(t = 2.800, P = 0.005), ^f^(χ^2^ = 8.416, P = 0.004), ^g^(t = 4.730, P<0.001), ^h^(χ^2^ = −91.588, P<0.001), ^i^(Z = −35.261, P<0.001).

Of the 453 with high CVD risk, 386 (85%) participated in the survey, 67 (15%) were not included because they were not reached after three attempts over one week. The median CVD risk of the 67 individuals who did not participate in the study was 26.3%, similar to that of the 386 who participated in the study. 62% of the participants were males, with an average age of 70 years. 294 (76%) had confirmed diagnosis of CVD or related disease: 37 (10%) had coronary heart disease, ischaemic or haemorrhagic cerebro-vascular disease, or heart attack, 269 (70%) had hypertension, 38 (10%) had diabetes, and 24 (6%) had hyperlipidaemia (Table 2). There were 92 (24%) subjects who had not been diagnosed any diseases. However, when their clinical details were compared with the national diagnostic guidelines [5], [24], 70 (76%) were suspects of hypertension, diabetes or hyperlipidaemia; and more females than males were likely to be suspects of CVD related disease. In total, 364 (94%) of individuals with risk of CVD 20% or higher, already had a diagnosed disease or were found to be suspects of having a CVD related disease when compared their recorded clinical information with the criteria in the national guidelines.

**Table 2:** Diagnosis of people with high risk of cardiovascular disease (CVD) in a rural primary care setting in Zhejiang, China.

	Male	Female	Total
Participants	240(62.2)	146(37.8)	386
Age	69	71	70
Those with a diagnosed disease:	166(69.2)	128(87.7)^a^	294(76.2)
Any CVD(%)	19(7.9)	18(12.3)	37(9.6)
Hypertension(%)	145(60.4)	124(84.9)^b^	269(69.7)
Diabetes(%)	26(10.8)	12(5.0)	38(9.8)
Hyperlipidaemia(%)	12(5.0)	12(8.2)	24(6.2)
Those without a diagnosed disease:	74(30.8)	18(12.3)	92(23.8)
Suspects of hypertension, diabetes orhyperlipidaemia (%)	52(70.3)	18(100.0)^c^	70(76.1)
Suspects of hypertension	48	18	66
Suspects of diabetes	9	0	9
Suspects of dislipidaemia	1	0	1
With or being suspects of a diagnosed CVD related disease (%)	218(90.8)	146(100.0)^d^	364(94.3)

Significantly higher than males: ^a^(χ^2^ = −17.123, P<0.001), ^b^(χ^2^ = −25.827, P<0.001), ^c^(Fisher’s exact test, P = 0.005), ^d^(χ^2^ = 14.192, P<0.001).

CVD include coronary heart disease, heart attack, and ischaemic or haemorrhagic cerebro-vascular disease.

**Figure 1:**
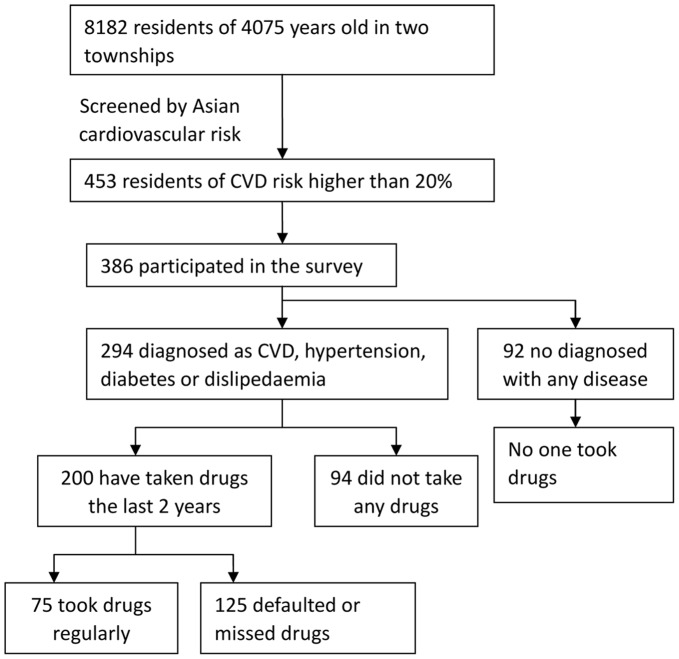
Number of people with high risk of cardiovascular disease (CVD) identified and their characteristics on diagnosis and drug taking in a rural primary care setting in Zhejiang, China.

Of the 294 high risk individuals with diagnosed CVD or related disease, 200 (68%) had ever taken drugs in the last two years. All the 92 high risk individuals not being diagnosed of any disease did not take drugs. At the time of survey, 101 were currently taking drugs, while 99 has stopped taking any drugs (Figure 1). Of the 200 patients who had ever taken drugs, 75 (38%) reported never missing their drugs, 88 (44%) reported missing at least one dose per week, while the remaining 37 (19%) reported having occasions of stopping treatment for at least a month. 165 (83%) reported following physician’s prescribed doses, while 28 (14%) reported taking less than the prescribed doses and 7 (14%) reported taking more than the prescribed doses. There were no significant difference between men and women regarding drug adherence.

Of the 200 who have taken drugs in the last two years, 76 (38%) reported using of calcium channel blocker drugs, 43 (22%) used angiotensin converting enzyme inhibitors (ACEi), 34 (17%) used thiazide diuretics and 6 (3%) used beta blockers. Only 8 (4%) used aspirin, 3 (1.5%) used statin and 1 (0.5%) used folic acid. In general 137 (69%) used any of the above drugs, while only 27 (13.5%) used a combination of any two or more of the above drugs. 69 (35%) reported ever used compounds containing modern (e.g. reserpine) and traditional Chinese medicines, such as kendir leaves and red sage root. More women (77%) than men (60%) used any of the above drugs; otherwise, there was no significant difference between genders regarding use of any particular type of drugs. The majority of respondents purchased their drugs from pharmacies, while 27% got prescriptions and purchased from township and county hospitals, and 24% had drugs purchased both from hospitals and pharmacies. The median cost of drugs was RMB 17 (USD2.66) per month. Women spent significantly more on CVD related drugs than men (RMB 20 vs. 12) (Table 3).

**Table 3:** Drug use of the 200 rural residents with high risk of cardiovascular disease (CVD) who were treated in a rural primary care setting in Zhejiang China.

	Males	Females	Total
Number	100	100	200
Drugs:			
Calcium channel blockers (%)	33(33.0)	43(43.0)	76(38.0)
Angiotensin converting enzyme inhibitors (%)	17(17.0)	26(26.0)	43(21.5)
Thiazide diuretics (%)	14(14.0)	20(20.0)	34(17.0)
Beta channel blockers (%)	3(3.0)	3(3.0)	6(3.0)
Statins (%)	1(1.0)	2(2.0)	3(1.5)
Aspirin (%)	5(5.0)	3(3.0)	8(4.0)
Folic acid (%)	0	1(1.0)	1(0.5)
Use any of the above drugs (%)	60(60.0)	77(77.0)^a^	137(68.5)
Use more than 1 of the above drugs (%)	13(13.0)	14(14.0)	27(13.5)
Use of compound tablets with traditionalChinese Medicine (%)	31(31.0)	38(38.0)	69(34.5)
Resources of drugs:			
Hospitals (%)	25(25.0)	28(28.0)	53(26.5)
Pharmacies (%)	53(53.0)	47(47.0)	100(50.0)
Both (%)	22(22.0)	25(25.0)	47(23.5)
Median costs of drugs per month (RMB)	12	20^b^	17

The proportion/average of females are significantly higher than that of males: ^a^(χ^2^ = −6.697, P = 0.010), ^b^(Z = −2.389, P = 0.017).

## Discussion

The study used a validated risk assessment tool to screen a rural population in China for having high risk of CVD. 5.5% of residents aged between 40 to 75 years (2.3% of the total population) were identified as having a CVD risk of 20% or more over within 8 years. The record review and interview found that the majority (76%) of those with a CVD risk of 20% or higher had been diagnosed: over 70% had hypertension, 10% had diabetes and 10% had a cardiovascular disease such as angina. Of those of high CVD risk not being diagnosed with a disease, the majority had elevated blood pressures, fasting blood glucose or lipid profile as compared to the national diagnostic guidelines [24]. Thus, they may be likely to be diagnosed as hypertension, diabetes (including pre-diabetes) or hyperlipidaemia after further clinical investigation. In the end, it is likely that over 94% of the high risk population should be diagnosed with CVD or related diseases.

Our findings suggest that this record review and CVD risk assessment strategy is useful in the detection of undiagnosed patients. Indeed, the majority of our undiagnosed high risk population was hypertensive or diabetic patients. The majority of residents who had hypertension or diabetes in this study in rural China were not aware of their diseases and/or not getting proper treatment. A national survey showed a 8.2% diabetes prevalence in rural areas in 2008; however, the rate of diagnosed diabetes was only 2.7% [25]. The 2002 national hypertension survey showed that 23% patients with hypertension were aware of it, 17% were on treatment, and 4% had their blood pressure under control [26].

Using this risk approach has cost-effective implication on the detection and treatment of CVD patients as well. A previous modeling study showed that applying risk assessment tools would result in treating the same number of patients as in the usual care as treating only patients identified with CVD related diseases. However, the risk screening approach, prioritising identification and treatment of those with high CVD risk, should prevent more CVD events and be more cost effective according to estimates in the modeling study [27].

In this study, we examined the use of highly effective drugs to prevent CVD events based on suggestions from systematic reviews and trials. It was found very few (26%) of the rural residents with high risk CVD were taking any drugs. A survey over 628 communities around the world found that less than one quarter of CVD patients ever took these preventative drugs, while over half of patients in developing countries did not use drugs at all [28]. The WHO PREMISE study reported over 70% of CVD patients in developing countries received aspirin, and around 20–40% received anti-hypertensive drugs and/or statins [29]. The WHO study was largely based on patients from teaching hospitals, thus resulting in a higher rates of drug intake compared with our study which was community based.

The reasons of low use of the highly effective drugs need further investigation, but are likely to include the limited knowledge of the doctors, belief in traditional therapies, non use of a CVD risk approach, and the cost of preventive drugs. However, these drugs are widely available in rural China at relatively affordable costs. Suppose that one takes the composition of nefidipine 30 mg, hydrochlorothiazide 50 mg, simvastatin 40 mg, aspirin 75 mg and folic acid 5 mg per day from the generic drugs, the total costs of drugs per month is RMB111 (USD17.3) in rural Zhejiang. The majority of the costs are from simvastatin, a statin. Without statin, the combination of two antihypertensives, aspirin and folate only costs as low as RMB6 (nearly US$1) per month. In addition, the rural health insurance in China covers 30% of drugs purchased from township hospitals. Paying the combination with statin may not be affordable, because the cost is much higher than the current RMB17 (USD6.3) per month found in our study, but paying a combination without statin should not be a problem. Another survey in northern China found hypertensive patients over-estimated the effect of drug treatment, and were willing to pay RMB 42 (USD6.6) per month for drug treatment if being informed having a CVD risk of 35% in 5 years [30]. Another possible reason is low awareness of the need of lifelong use of the preventative drugs by doctors in rural practice, so the highly effective drugs are simply not prescribed. For those taking drugs, 44% of participants in this study reported missing at least one dose per week, while 19% reported having occasions of stopping treatment for at least a month. Our findings suggest that adherence to drugs could be a challenge in the CVD prevention and treatment, echoing the study elsewhere [26], [28]. Adherence support strategies can be learned from tuberculosis control using treatment supporters and reminders [31], [32], though CVD is much challenging because it needs life-long treatment.

The current health reform in China makes a CVD risk reduction strategy feasible. The resident health records can provide the basis to apply risk assessment and identify those with high risk of CVD. Primary care facilities, including township hospitals, village clinics and community health centres, are strengthened with recent government policy investment in public health activities. Chronic disease control has become a responsibility of doctors and nurses at the primary care facilities. Zhejiang provincial government has designated outpatient doctors in township hospitals as “family doctors”. One family doctor is responsible for acute and preventative care, including chronic disease control, for an average population of 1500. Currently in China CVD risk reduction is not generally practiced [7]. Based on this, we would suggest a holistic CVD risk reduction approach, which should include screening patients/records, the use of highly effective drugs, adherence support and healthy lifestyle support. This package can be designed to be implemented by primary care health workers in rural China [33].

The study has several limitations. First, the Asian Equation estimates CVD events in 8 years while most other predictive equations estimate for 10 years. Using a similar cut-off of 20% as high CVD risk, the number of high risk people with CVD identified by the Asian Equation may be lower than that identified using other equations. However, the Asian equation has better predictive value on both ischaemic and hamoerrhagic CVD events compared with other equations in Chinese population. Second, samples are taken from two townships in rural Zhejiang, thus, the results cannot be extrapolated further. Third, information of the resident health records were collected by doctors and nurses from township hospitals. The accuracy may not be complete; however, all doctors and nurses involved in building up the health record were trained based on a provincial guideline, and all diagnosis of CVD, hypertension, diabetes and hyperlipedaemia were checked against their previous medical records. Fourth, a further caution in interpretation is that 67 (15%) of the high risk population did not participate in the survey, though their CVD risk was not different from those who participated in the survey. Furthermore we did not collect information in the survey regarding daily salt intake, exercise or any healthy lifestyle advice given by their doctors or nurses.

### Conclusion

By examining the current situation of rural population with high risk of CVD in China, the study found that the majority of the high risk population had already been diagnosed with a CVD related disease. However, only a quarter of these were currently taking any drugs, and less still took highly effective drugs to prevent CVD. We suggest a community-based CVD risk reduction approach, designed to be implementable within the current China public health and rural health insurance reforms, in so doing improve care and reduce CVD events at the primary care level.
